# A Novel Parallel Processing Model for Noise Reduction and Temperature Compensation of MEMS Gyroscope

**DOI:** 10.3390/mi12111285

**Published:** 2021-10-21

**Authors:** Qi Cai, Fanjing Zhao, Qiang Kang, Zhaoqian Luo, Duo Hu, Jiwen Liu, Huiliang Cao

**Affiliations:** 1 Science and Technology on Electronic Test & Measurement Laboratory, North University of China, Taiyuan 030051, China; c18434363610@163.com; 2 School of Instrument and Electronics, North University of China, Taiyuan 030051, China; 1805004128@st.nuc.edu.cn (Z.L.); 1806034238@st.nuc.edu.cn (D.H.); liujiwen200143@163.com (J.L.); 3 NORINCO GROUP Test and Measurement Academy, Planning Division, Huayin 714200, China; kangqiang051@163.com

**Keywords:** MEMS gyroscope, temperature compensation, multi-objective particle swarm optimization (MOPSO), variational modal decomposition (VMD), beetle antennae search algorithm (BAS), Elman neural network (Elman NN)

## Abstract

To eliminate the noise and temperature drift in an Micro-Electro-Mechanical Systems (MEMS) gyroscope’s output signal for improving measurement accuracy, a parallel processing model based on Multi-objective particle swarm optimization based on variational modal decomposition-time-frequency peak filter (MOVMD–TFPF) and Beetle antennae search algorithm- Elman neural network (BAS–Elman NN) is established. Firstly, variational mode decomposition (VMD) is optimized by multi-objective particle swarm optimization (MOPSO); then, the best decomposition parameters [*k*_best_,*a*_best_] can be obtained. Secondly, the gyroscope output signals are decomposed by VMD optimized by MOPSO (MOVMD); then, the intrinsic mode functions (IMFs) obtained after decomposition are classified into a noise segment, mixed segment, and drift segment by sample entropy (SE). According to the idea of a parallel model, the noise segment can be discarded directly, the mixed segment is denoised by time-frequency peak filtering (TFPF), and the drift segment is compensated at the same time. In the compensation part, the beetle antennae search algorithm (BAS) is adopted to optimize the network parameters of the Elman neural network (Elman NN). Subsequently, the double-input/single-output temperature compensation model based on the BAS-Elman NN is established to compensate the drift segment, and these processed segments are reconstructed to form the final gyroscope output signal. Experimental results demonstrate the superiority of this parallel processing model; the angle random walk of the compensated gyroscope output is decreased from 0.531076 to 5.22502 × 10^−3^°/h/√Hz, and its bias stability is decreased from 32.7364°/h to 0.140403°/h, respectively.

## 1. Introduction

Thanks to the emergence and progress of Micro-Electro-Mechanical Systems (MEMS) technology, the research and application of MEMS inertial devices have attracted extensive attention [1,2,3,4,5]. As one of the outstanding representatives, MEMS gyroscopes are used in aerospace, consumer electronics and other high-precision control and measurement fields widely because of low power consumption, small size, easy integration, high reliability, and high precision [6,7]. Although the MEMS gyroscope has many superior advantages, its performance is affected by the temperature drift due to its manufacturing process and inherent structural characteristics, so how to effectively eliminate the influence on the gyroscope has become a research hotspot [8,9,10,11,12,13,14,15,16,17,18].

In general, the methods to reduce the influence of temperature drift on gyroscope performance are classified into two categories: hardware temperature compensation and software temperature compensation. The hardware temperature compensation method is used to reduce temperature error from the perspective of device optimization, including improving the manufacturing process and optimizing the sensor structure and circuit control system. Many domestic and foreign experts have proposed different hardware compensation schemes, which have made good progress [9,10,11,12,13,14,15,16]. Cui et al. [9] analyzed the driving modes and sensing modes of the gyroscope, and they designed a model for temperature compensation according to the vibration characteristics of the driven mode using a variable temperature resistor. Fu et al. [10] designed a constant transconductance high linear amplifier that achieves low phase drift and low amplitude drift interface circuits over all temperature ranges. Guo et al. [11] designed a resonant MEMS gyroscope with a self-temperature compensation function to eliminate temperature errors, in which the gyroscope’s sensing unit is composed of four double-ended tuning forks with symmetrical distribution. Based on the bipolar temperature compensation method, the sensing mode closed-loop method for the gyroscope is introduced by Cao et al. [12]. However, hardware compensation has a long research and development cycle, high consumption, and is not easy to realize.

Another method is the software compensation; the corresponding relationship between the temperature input and the gyroscope output is obtained by establishing the temperature compensation model. In it, a set of experimental temperature and gyroscope output obtained in advance are trained as input and output to establish the model. The temperature compensation model has two problems to solve: one is the noise in the high-frequency component, and the other is the drift caused by temperature change in the low-frequency component. Due to the different processing methods, the temperature compensation model can be divided into serial processing and parallel processing models. Serial processing is to denoise the entire output signal first and then establish the compensation model to eliminate the temperature drift. The method of first denoising and then compensation will lead to the loss of useful signals in the original signal, leading to unsatisfactory compensation results [17,18,19,20,21,22].

Different from the serial processing model, the parallel processing model is used to extract the noise component and drift component of the signal and then carry out noise reduction and compensation processing respectively at the same time and get the final signal through reconstruction. As two representative adaptive decomposition algorithms, empirical mode decomposition (EMD) and variational mode decomposition (VMD) are used in the engineering fields widely. However, mode mixing affects the decomposition effect of EMD, and VMD has improved the mode mixing by constructing and solving the variational model [23].

Unfortunately, the proper decomposition parameters of VMD should be selected before using. When the decomposition number *k* is set unreasonably, over-decomposition or under-decomposition will occur. On the other hand, the larger the penalty factor α, the wider the bandwidth of the intrinsic mode function, and vice versa, which affects the decomposition accuracy of VMD [24]. Therefore, it is significant to select the appropriate VMD decomposition parameters [*k*,*a*]. Thanks to the emergence of intelligent algorithms such as swarm optimization algorithms and neural networks, many scholars have used these algorithms to optimize the VMD [25,26,27]. These optimization algorithms realize the purpose of optimization by constructing the single objective function, which only considers the problem in one aspect, while the multi-objective optimization algorithm comprehensively considers the optimization of the target from many aspects and can obtain the global optimal characteristics. As one of the multi-objective optimization algorithms, multi-objective particle swarm optimization (MOPSO) [28] has been successfully applied to the engineering fields in view of its simple theory, fast convergence, strong global optimization ability, flexible parameter adjustment mechanism, and other characteristics.

In addition, the establishment of a high-precision compensation model is an important link in the parallel processing model; intelligent algorithms are competent at establishing corresponding relationships between temperature and gyroscope output. For example, BP neural network, extreme learning machine (ELM), support vector machine (SVM), and the improvement of these algorithms have been widely used in temperature compensation and achieved good results [17,18,19,20,21,29]. Among them, Elman neural network (Elman NN) is a dynamic recursive network with feedback; the increase in feedback layer makes the network have time-varying adaptive characteristics, thus increasing the global stability of the network. As an improvement of BP neural network, it also inherits its disadvantages to some extent, which will lead to local optimization [22]. To improve the processing accuracy of Elman NN, the beetle antennae search algorithm (BAS) is adopted to optimize the Elman NN.

Therefore, the parallel processing model for eliminating noise and temperature drift based on MOVMD–TFPF and BAS–Elman NN is put forward. Firstly, MOPSO is adopted to determine the optimal decomposition parameters of VMD; then, the gyroscope output signal is decomposed by MOVMD. Secondly, the intrinsic mode functions (IMFs) obtained after decomposition are classified by sample entropy (SE) into a noise segment, mixed segment, and drift segment. Then, the noise segment is discarded directly, the mixed segment is denoised by TFPF, and the drift segment is compensated by double-input/single-output temperature compensation model based on the BAS–Elman NN at the same time. Finally, the final gyroscope output signal can be get through reconstructing the processed segments, details of the algorithm theory, experimental process, and comparative analysis are given in the following sections.

## 2. Introduction of Dual-Mass MEMS Gyroscope

### 2.1. Dual-Mass MEMS Gyroscope’s Structure

**Figure 1:**
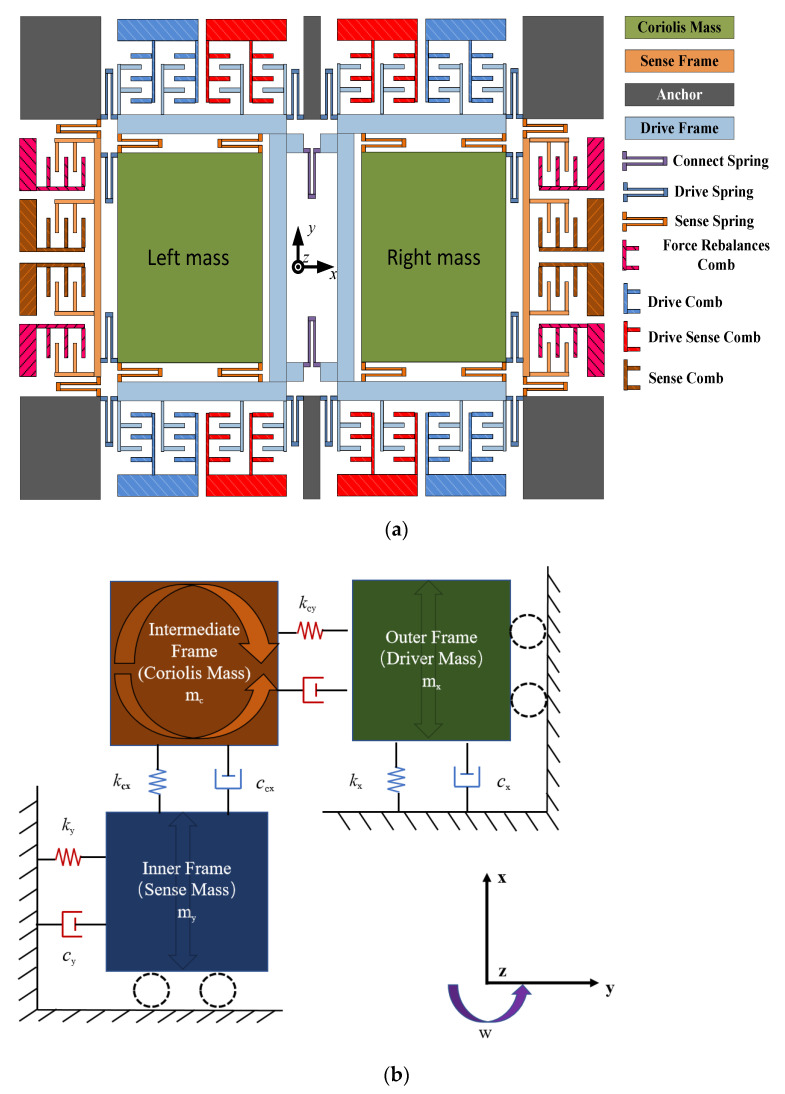
(**a**) The gyroscope’ structure; (**b**) The gyroscope’ mechanical model.

A dual-mass MEMS gyroscope is adopted for the temperature experiments in this article, and the overall structure is shown in Figure 1 [30]. When the gyroscope works, there are two main modes: the drive mode and sense mode. Between them, the drive comb, drive spring, and other parts constitute the drive mode, which is moving on the X-axis direction; the sense comb, sense spring, and other sections compose the sense mode, which is moving along the Y-axis. In Figure 1, the two sensitive masses (left and right masses) are the parts of both modes, which vibrate along the negative direction of the X-axis. In addition, the two modes of the gyroscope are isolated from each other to avoid the generation of coupling displacement. When the angular velocity Ωz is input around the Z-axis, the Coriolis force generated by the vibrating mass is passed to the frame along the Y-axis and later examined through a monitoring electric circuit.

**Figure 2:**
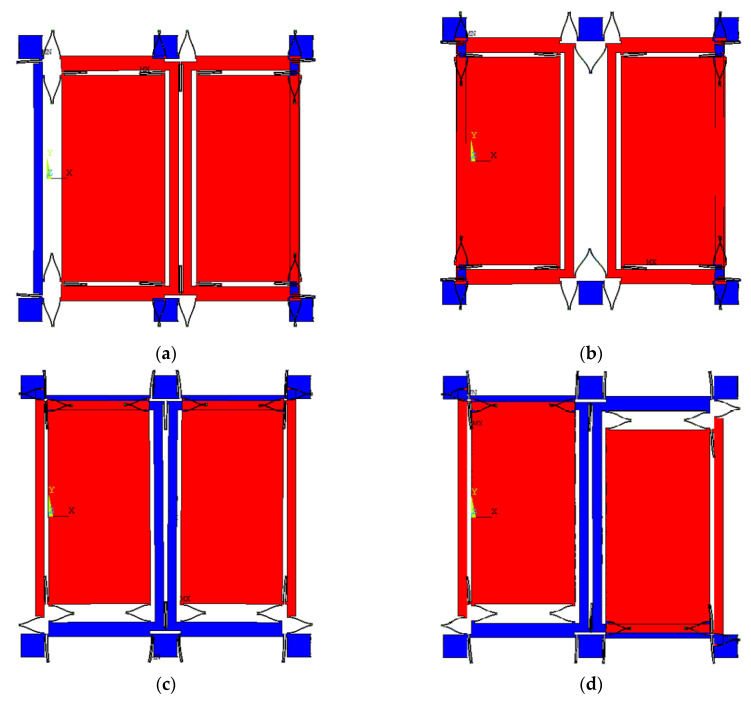
The four working modes of gyroscope. (**a**–**d**) are the four modes of the gyroscope respectively, and their corresponding frequencies are *w*_1_ = 2623 × 2π rad/s, *w*_2_ =3342 × 2π rad/s; *w*_3_ = 3468 × 2π rad/s; *w*_4_ =3484 × 2π rad/s.

The gyroscope works on the basis of tuning fork theory. The U-type connecting spring is connected to the dual-drive mass block, and the drive spring is connected to the dual-sense mass block. In order to analyze the gyroscope’s working modes, Ansys Software (ANSYS, Pittsburgh, PA, USA) is adopted to simulate these working modes; the results are shown in Figure 2 [30]. It can be found that the frequency gap between the first and the fourth working mode is large, which is more than 1000 Hz, and the fourth mode of the gyroscope has a quality factor of more than 2000, so the fourth mode is the gyroscope drive mode. Figure 2a–d show the analysis results of the gyroscope’s four working modes in turn. The drive in-phase mode is the first mode, in which the vibration direction of the double mass of the gyroscope is consistent with the X-axis. The second mode is the sensing in-phase mode, in which the two masses simultaneously vibrate consistent with the Y-axis direction. The third mode is the sensing anti-phase mode; in this mode, the two mass blocks oscillate in the opposite direction of the Y-axis. The fourth mode is the drive anti-phase mode, in which the two mass blocks of the gyroscope oscillate in the contrary direction to the Y-axis. In addition, the resonant frequencies of these four modes are 2623 Hz, 3342 Hz, 3468 Hz, and 3484 Hz. The fourth mode is not only the drive anti-phase mode but also the true driving mode. Combined with the above analysis, it can be analyzed that the two mass blocks of the gyroscope have two degrees of freedom (X-axis and Y-axis), while the drive mode and frame has only a single degree of freedom (X-axis or Y-axis).

### 2.2. Gyroscope’s Periphery Circuit

**Figure 3:**
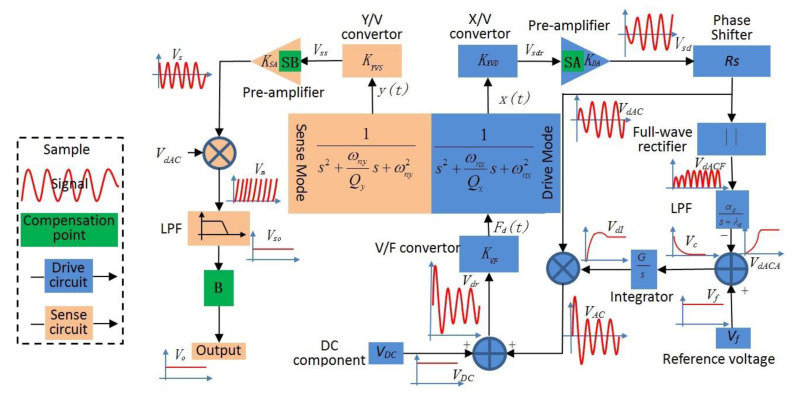
Gyroscope’s peripheral circuit and the signal flow.

In Figure 3 [31], because the drive circuit is controlled by an AGC closed-loop, the drive mode can maintain constant amplitude motion at the resonant frequency. Then, the sense circuit detects the displacement of the sense mode and completes signal processing.

For the drive loop, the drive sense comb is used to measure the shift of the drive frame *x*(*t*), and the *x*(*t*) is then converted into a voltage signal *V_sdr_* by a linear transformation of the X/V converter, which is amplified into *V_sd_*. To satisfy the phase required for the AC drive signal *V_dAC_* = *V_dACA_*Sin(*w_d_t*) as much as possible, the phase of *V_sd_* should be delayed by 90°. After that, the signal *V_dACA_* is extracted through a full-wave rectifier and low-pass filter, and the extracted signal is contrasted to the reference voltage *V_f_* at the same time. Then, the control signal *V_dI_* can be obtained when the output signal of the comparator passes through the integrator controller, and *V_dI_* is used to modulate *V_dAC_* to get the drive AC signal *V_AC_*_._ Finally, the DC signal *V_DC_* is driven to superimpose with *V_AC_* to form a force that can create an excitation for the drive mode.

For the sense circuit, it is open-loop and uses the same interface as the drive circuit.

The motion signals of the left and right sensitive mass blocks are captured by the differential detection amplifier, and then, the second differential amplifier is adopted to process the output signal to generate sense motion signal *V_s_*. Then, *V_s_* is demodulated by *V_dAC_* and then denoised by the low-pass filter to obtain the final sensitive motion signal *V_so_*; for the signal *V_so_*, the compensation module “B” or compensation algorithms can be added to compensate it and finally get the compensation signal V_o_.

## 3. Algorithms and Models

### 3.1. Variational Mode Decomposition (VMD)

The VMD is an effective decomposition method for processing nonstationary signals. Different from EMD, which decomposes complex signals by recursion-filter decomposition, VMD decomposes complex signals by non-recursive decomposition and constructing a variational model. The optimal solution of the variational model is searched through cyclic iterative processing, which means that the complex signals are decomposed into many IMFs, and each IMF has the center frequency and limited bandwidth. This enables VMD to avoid the mode aliasing phenomenon existing in EMD and has better noise robustness. The decomposition principle of VMD is briefly described as follows [32].

(1) The construction of constrained variational model.

Suppose that any complex signal *y*(*t*) is decomposed into *k* IMFs {*u*_k_(*t*)} = {*u*_1_(*t*), *u*_2_(*t*), *u*_3_(*t*), …, *u_k_*(*t*)} with a center frequency and finite bandwidth; the variational model is constructed to seek the optimal mode functions so as to minimize the sum of estimated bandwidths of all intrinsic mode functions. The variational model is constructed as follows:

a. Hilbert transformation is performed on the obtained mode functions to obtain their analytic signals; the purpose is to get the unilateral spectrum of each mode function.

(1) $$\left[\sigma (t)+\frac{j}{\pi t}]*u_{k}(t\right)$$

b. To obtain the constrained variational model, the center frequency of each modal analytical signal obtained in Equation (1) is initialized; then, the square norm of the demodulation signal gradient is calculated, and the bandwidth of each IMF is estimated:

(2) $$\{\begin{array}{c} \underset{\{u_{k},\theta_{k}\}}{\min}\{\sum_{k}{\left\|\partial_{t}\left[\left(\sigma (t)+\frac{j}{\pi t})u_{k}(t\right)\right]e^{-jw_{k}t}\right\|}_{2}^{2}\}\\ s.t\sum_{k}u_{k}=y(t) \end{array}$$

where {*θ*_k_} = {*θ*_1_, *θ*_2_, …, *θ_k_*} is the collection of central frequencies of each IMF.

(2) The solution of the constrained variational model.

a. To simplify the constrained variational model, the unconstrained variational model is constructed by constructing an extended Lagrangian expression. In Equation (3), *a* and *λ* are the penalty factor and Lagrangian multiplication operator.

(3) $$\begin{array}{l} L\left(\{u_{k}\},\{\theta_{k}\},\lambda \right)=\alpha {\sum_{k}\|\partial_{t}\left[\left(\sigma (t)+\frac{j}{\pi t}\right)*u_{k}(t)\right]e^{-j\theta_{k}t}\|}_{2}^{2}\\ \;{\|y(t)-\sum_{k}u_{k}(t)\|}_{2}^{2}+\langle \lambda (t),y(t)-\sum_{k}u_{k}(t)\rangle \end{array}$$

b. The corresponding extremum solution can be obtained by transforming the Lagrangian function obtained by Equation (3) in the time-frequency domain. The expressions for *u_k_* and *θ_k_* are as follows, respectively:

(4) $$u_{k}^{n+1}(\theta )=\frac{y-\sum_{i\neq k}^{}u_{i}(\theta )+\frac{\lambda (\theta )}{2}}{1+2\alpha {\left(\theta -\theta_{k}\right)}^{2}}$$

(5) $$\theta_{k}^{n+1}=\frac{\int_{0}^{\infty }\theta |u_{k}(\theta ){|}^{2}d\theta }{\int_{0}^{\infty }\left|u_{k}(\theta \right){|}^{2}d\theta }$$

c. The alternating direction multiplier algorithm is adopted to update the parameters *u_k_^n+^*^1^, *θ_k_^n+^*^1^, and *λ^n+^*^1^, and the updated formula of *λ^n+^*^1^ is:

(6) $$\lambda^{n+1}(\theta )\leftarrow \lambda^{n}(\theta )+\tau [y(\theta )-\sum_{k}u_{k}^{n+1}(\theta )]$$

In Equation (6), *τ* is the time constant factor, which affects the update of *λ*. If the accuracy is not strictly required, the update can be avoided. In this case, *τ* = 0.

d. When the condition of Equation (7) is satisfied, the iteration stops, and *k* intrinsic mode functions are output. Otherwise, the iteration continues by following the formulas above.

(7) $$\sum_{k=1}^{K}\frac{{\|u_{k}^{n+1}(\theta )-u_{k}^{n}(\theta )\|}_{2}^{2}}{{\|u_{k}^{n}(\theta )\|}_{2}^{2}}<\varepsilon$$

### 3.2. Multi-Objective Particle Swarm Optimization

The MOPSO algorithm is a widely used intelligent algorithm, which combines the particle swarm optimization (PSO) and the grid algorithm; it advances the original single target optimization to multiple targets. It is based on the predation behavior of birds, and it has excellent convergence speed and good overall search ability. The reasonable selection of multiple fitness functions is also the key to MOPSO; fuzzy entropy (FE) and permutation entropy (PE) are selected as fitness functions of MOPSO to optimize the VMD in this article.

Fuzzy entropy (FE) is an algorithm that can reflect the complex components of the measured nonlinear signal; the more sparse the signal is, the greater the fuzzy entropy. At the same time, FE is also an improvement on the approximate entropy; it is less dependent on time series and more robust to noise-containing signals, and the brief introduction to fuzzy entropy (FE) is as follows [33]:

Step 1. Reconstruct phase space.

For the time series {*s(p)*, 1 ≤ *p* ≤ N}, phase space reconstruction is carried out to obtain m-dimensional vectors:

(8) $$\begin{array}{l} X_{p}^{m}=\{s(p),s(p+1),\ldots ,s(p+m-1)\}-s_{0}(p)\\ \begin{array}{cc} & p=1,2,\ldots ,N-m+1 \end{array} \end{array}$$

Here, *X_p_^m^* is m consecutive values of s starting at the *p*th point and subtracting the mean *s_0_*(*p*):

(9) $$s_{0}(p)=\frac{1}{m}\sum_{q=0}^{m-1}s(p+q)$$

Step 2. Define the distance between vectors.

*D^m^_pq_* is the maximum difference between vector *X^m^_p_* and *X^m^_q_*, namely:

(10) $$\begin{array}{l} D_{pq}^{m}=d\left[X_{p}^{m},X_{q}^{m}\right]=\underset{k\in (0,m-1)}{\max}\{|[s(p+k)-s_{0}(p)]-[s(k+q)-s_{0}(q)]|\}\\ \;(p,q=1,2,\ldots ,N-m,p\neq q) \end{array}$$

Step 3. Compute the membership degree between vectors.

The membership degree of vector *X^m^_p_* and *X^m^_q_* is defined as *µ*(*d^m^_pq_*,θ,*ω*), which is:

(11) $$D_{pq}^{m}=\mu (d_{pq}^{m},\theta ,\omega )=e^{-{\left(\frac{d_{pq}^{m}}{\omega }\right)}^{\theta }}$$

In the formula, the fuzzy function is defined as *µ*(*d^m^_pq_*,θ,*ω*), which is an exponential function, and the gradient and width of its boundary are denoted as *θ* and *ω*.

Step 4. Define the function.

(12) $$\Phi^{m}(\theta ,\omega )=\frac{1}{N-m}\sum_{p=1}^{N-m}(\frac{1}{N-M-1}\sum_{q=1,q\neq p}^{N-m}D_{pq}^{m})$$

Similarly, for *m* + 1 dimension vector, repeat the Formulas (8)–(11); then, the formula can be obtained:

(13) $$\Phi^{m+1}(\theta ,\omega )=\frac{1}{N-m}\sum_{p=1}^{N-m}(\frac{1}{N-M-1}\sum_{q=1,q\neq p}^{N-m}D_{pq}^{m+1})$$

Step 5. Define fuzzy entropy.

(14) $$F_{E}(m,\theta ,\omega )=\underset{N\to \infty }{\lim}[\ln\Phi^{m}(\theta ,\omega )-\ln\Phi^{m+1}(\theta ,\omega )]$$

When *N* is a finite value, Equation (14) is simplified as follows:

(15) $$F_{E}(m,\theta ,\omega )=\ln\Phi^{m}(\theta ,\omega )-\ln\Phi^{m+1}(\theta ,\omega )$$

Another fitness function permutation entropy (PE) is introduced as follows.

PE is first proposed by Bandt et al. [34], which can be used to calculate the complexity and randomness of complex signals. PE is used to measure the noise level of the signal in this paper, and the principle of PE is as follows:

Step 1. Reconstruct phase space.

For the time series {*u*(*j*), 1 ≤ *j* ≤ N}, phase space reconstruction is carried out to get a phase sequence:

(16) $$R=\left[\begin{array}{l} R(1)\\ R(2)\\ \vdots \\ R(l)\\ \vdots \\ R(k) \end{array}\right]=\left[\begin{array}{l} \begin{array}{cc} u(1) & u(1+\omega )\begin{array}{cc} \ldots & u(1+(e-1)\omega ) \end{array} \end{array}\\ \begin{array}{cc} u(2) & u(2+\omega )\begin{array}{cc} \ldots & u(2+(e-1)\omega ) \end{array} \end{array}\\ \begin{array}{cc} \vdots & \begin{array}{cc} \;\vdots & \begin{array}{cc} \;\vdots & \end{array} \end{array} \end{array}\\ \begin{array}{cc} u(l) & u(l+\omega )\begin{array}{cc} \ldots & u(l+(e-1)\omega ) \end{array} \end{array}\\ \begin{array}{cc} \vdots & \begin{array}{cc} \;\vdots & \begin{array}{cc} \;\vdots & \end{array} \end{array} \end{array}\\ \begin{array}{cc} u(k) & u(k+\omega )\begin{array}{cc} \ldots & u(k+(e-1)\omega ) \end{array} \end{array} \end{array}\right]$$

Here, *e* is the embedded dimension, *k + (e − 1)ω = n*, *R*(*l*) represents the reconstructed vector, there are a total of *k* reconstruction vectors, and the delay time is denoted as *ω*.

Step 2. Rearrange the reconstructed vectors.

Each reconstructed vector is rearranged according to the size; then, the column indexes of elements in the vector are obtained to form a set of symbol sequences {*h*_1_*,h*_2_*,h*_3_*...*,*h_m_*}, namely:

(17) $$s(l+(h_{1}-1)\omega )\leq \ldots \leq s(l+(h_{m}-1)\omega )$$

When *h_p_ < h_q_*, that is:

(18) $$s(l-(h_{p}-1)\omega )\leq s(l-(h_{q}-1)\omega )$$

Step 3. The calculation and normalization.

After the rearrangement, calculate the probability of each symbol sequence and denote them as *P*_1_, *P*_2_*...*, *P_r_*, and the calculation formula of permutation entropy is:

(19) $$H_{p}(e)=-\sum_{n=1}^{e}p_{k}\ln p_{k}$$

The maximum of permutation entropy is ln *e*!; normalize the permutation entropy, that is:

(20) $$H_{p}=\frac{H_{p}(e)}{\ln e!}\begin{array}{ccc} & & H_{p}\in [0,1] \end{array}$$

The normalized permutation entropy can be used to calculate the complexity and randomness of complex signals: that is, the larger the permutation entropy is, the higher the complexity and randomness of complex signals will be, and vice versa.

The brief description of the steps of the MOPSO algorithm is as follows [24].

A. Firstly, the key parameters of MOPSO are set, including the total particle number *N_P_*, maximum iteration number *M*, save set size *N_R_*, etc. The number of particles affects the searching ability of MOPSO. When the number of particles is set too large, the algorithm has a good global searching ability, but it will affect the speed of the algorithm.

B. Initialize the particle swarm *P*_1_: The position *P*(*j*) of each particle is randomly initialized, while its velocity *v*(*j*) is set to zero. The fuzzy entropy and permutation entropy are adopted as fitness functions to evaluate each particle. When the fitness values are smaller, the corresponding VMD decomposition parameters are better. Meanwhile, the non-inferior solution in *P*_1_ is stored in the save set *N_P_*.

C. Update the individual best particle *P_best_* and the global best particle *G_best_*, use the adaptive grid method to find the global optimal particle *G_best_*, and continuously evolve to generate the next generation particle population; perform the following steps before the save set reaches the maximum:

(1). Calculate the density information of the particles in the save set, divide the target space into small areas by the grid, and measure the density by the number of particles in each area.

(2). The historical optimal position is updated when the particle’s current position is better than the best position of the individual history. Then, the global optimal particle Gbest is selected for the particles in the population, and the selection is based on the density information of the particles. Specifically, for a particle in the save set, the lower the density value, the greater the probability of selection.

(3). Update the position and velocity of each particle. In addition, the particles search for the optimal solution under the leadership of *G_best_* and *P_best_*:

(21) $$v_{i,d+1}^{j}=\mu (wv_{i,d}^{j}+c_{1}R_{1}(P_{i,d}^{j}-x_{i,d}^{j})+c_{2}R_{2}(G_{i,d}^{j}-x_{i,d}^{j}))$$

(22) $$x_{i,d+1}^{j}=x_{i,d}^{j}+v_{i,d+1}^{j}$$

where *d* represents the algebra of the current particle evolution, *i* represents the current evolutionary particle, *c*_1_ and *c*_2_ are the learning factors, *μ* is the contraction factor, *R*_1_ and *R*_2_ are the random numbers in the interval [0, 1], and $P_{i,d}^{j}$ and $G_{i,d}^{j}$ represent the value of the *j*-th decision vector of *P_best_* and *G_best_* of the particle, respectively. The save set is updated; after the evolution of the new generation group *P_d+_*_1_, the non-inferior solutions in *P_d+_*_1_ are saved to the save set.

D. If the number of particles in the save set exceeds the set maximum value, the individuals in the dense range are replaced, and the individuals in the sparse range are retained to maintain the size of the save set. For a grid with more than one particle, calculate the number of particles ND to be deleted in the grid according to Formula (23), and then randomly delete the ND particles in the grid.

(23) $$ND=Int(\frac{\left|A_{t+1}-\bar{N}\right|}{\left|A_{t+1}\right|}\times Grid[k,2]+0.5)$$

where *A_t_* is the quantity of particles in the save set, and Gird[*k*] is the quantity of particles in grid *k*.

E. When the stop condition is reached, the iteration is stopped, the particle information in the storage set is output, and the optimal decomposition parameters [*k_best_*,*a_best_*] of VMD can be obtained.

### 3.3. Time-Frequency Peak Filtering (TFPF)

TFPF is a noise elimination technology introduced by Mesbah et al. [35]. Thanks to its ability to extract effective signals in a noisy environment, it has been applied widely in many engineering fields.

The TFPF algorithm is mainly based on Wigner–Ville distribution (WVD) and instantaneous frequency estimation theory to filter and de-noise signals. Due to its good time-frequency focusing property, WVD is widely used in engineering. However, when WVD processes multi-component signals, the resolution of time-frequency distribution of signals will be reduced due to the generation of cross terms, which leads to the weakening of time-frequency focusing of WVD. To suppress the cross terms in TFPF, the pseudo-Wiener–Ville distribution is adopted. According to the principle of TFPF, it is necessary to encode the noisy signal to make it become the analytic signal of instantaneous frequency firstly, and the estimated value of the effective signal can be obtained through estimating its instantaneous frequency.

The gyroscope output signal is mixed with noise:

(24) y(t)=x(t)+n(t)

where *x*(*t*) and *n*(*t*) represent the useful signal and noise in the gyroscope output respectively, and the steps of TFPF are as follows [36]:

Step 1. Through frequency modulation of signal *y*(*t*) containing noise, the analytic signal *z*(*t*) can be obtained:

(25) $$z(t)=e^{j2\pi \mu \int_{0}^{t}y(\lambda )d\lambda }$$

Here, *µ* is the frequency modulation index.

Step 2. The pseudo-Wigner–Ville distribution spectrum of the analytic signal *z*(*t*) is calculated:

(26) $$PW_{2}(t,f)=\int_{-\infty }^{\infty }h(\tau )z(t+\frac{\tau }{2})z^{*}(t-\frac{\tau }{2})e^{-j2\pi ft}d\tau$$

where *t* stands for time, *τ* stands for integral variable, *f* stands for frequency, z^*^ stands for the conjugated operator of *z*, the window function is denoted as *h(τ)*, and the window length is a tradeoff parameter of TFPF.

Step 3. According to the maximum likelihood estimation principle, the peak value of the PWVD distribution spectrum of the analytic signal is calculated as the instantaneous frequency estimation of the analytic signal, and the amplitude estimation of the original effective signal can be obtained:

(27) $$f_{z}(t)=\frac{\arg\max[PW_{z}(t,f)]}{\mu }$$

### 3.4. Temperature Compensation Model Based on BAS–Elman NN

#### 3.4.1. The Framework of Compensation Model

**Figure 4:**
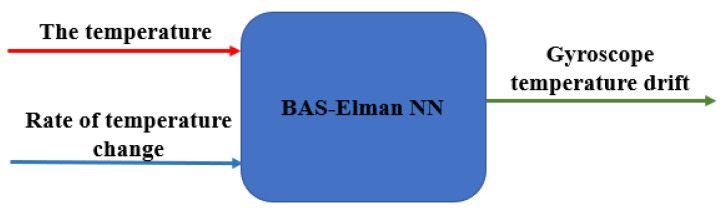
Temperature compensation model framework.

In general, the temperature compensation model is established to study the relationship between temperature and temperature drift in the gyroscope output. Unfortunately, the general temperature compensation models only consider the temperature itself, which leads to the unsatisfactory accuracy of the compensation model. To improve the model accuracy, the temperature change rate is added in this paper to describe the temperature field. Assume that the observed value of the gyroscope temperature field is *M*:

(28) $$M=\left[\begin{array}{c} T\\ \Delta T \end{array}\right]$$

where *T* represents the temperature, and ∆*T* describes the rate of temperature change. After obtaining the temperature field and combining with the temperature drift in the gyroscope, the mapping model is established:

(29) $$S=R(M)=R\left\{{\left[T_{},\Delta T_{}\right]}^{\prime }\right\}$$

where *S* is temperature drift and *R*(.) is the prediction function, which is a multi-input/single-output model established by neural network. The model framework is shown in Figure 4, and the Elman neural network optimized by the beetle antennae search algorithm is used as the learner.

#### 3.4.2. Beetle Antennae Search Algorithm (BAS)

**Figure 5:**
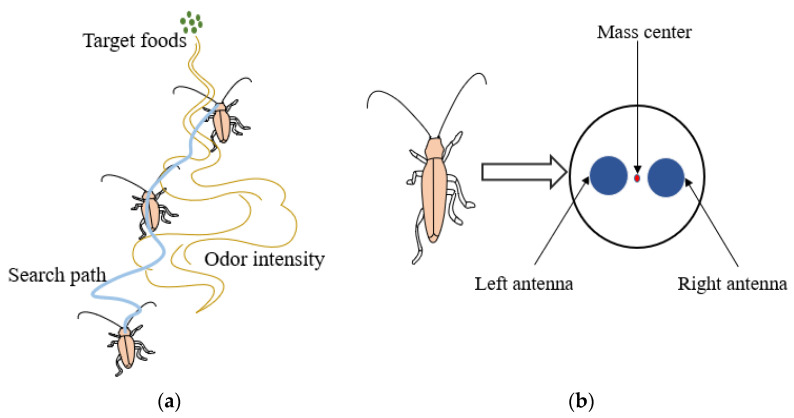
(**a**) Foraging behavior of the long-antenna beetle. (**b**) Simplified model of beetle.

BAS is an efficient intelligent optimization algorithm that is inspired by the principle of beetles foraging. It does not need to know the specific form and gradient information of the function, and it only needs one individual in the target search. Due to its simple and flexible drifts, avoiding local optimal solutions and being suitable for multi-latitude search, BAS has shown wide applicability and high efficiency in solving complex optimization problems [37,38,39]. The basic principle of the BAS algorithm is that when the beetle is looking for food, it will randomly explore the information intensity in the nearby space by swinging the long antennae on the sides of its body. When one antenna detects a higher information intensity than the other, the beetle will move to the side with the higher information intensity, and it will continue to explore the information intensity at random until it finds food. The beetle foraging diagram and simplified beetle model are shown in Figure 5a,b, respectively [40].

As shown in Figure 5b, the BAS algorithm simplifies the individual beetle and regards it as a particle that can sense the left and right direction. Since the direction of the beetle head is random, the right antennae of the beetle will generate a direction vector pointing to the left antennae to indicate the movement direction of the beetle. The steps of BAS algorithm are as follows [37,38]:

1. Create a random direction vector that represents the search behavior of beetle antennae:

(30) $$\vec{S}=\frac{rands(n,1)}{\left\|rands(n,1)\right\|}$$

*Rands*(.) is the random function, and *n* is the dimension of the search space.

2. Create the spatial coordinates of the antennae:

(31) $$\{\begin{array}{c} x_{lt}=x^{t}-d^{t}\vec{S}\\ x_{rt}=x^{t}+d^{t}\vec{S} \end{array}$$

In Formula (31), *x^t^* is the beetle’s spatial position at the *t*-th search, *x_lt_* and *x_rt_* respectively represent the spatial positions of the beetle’s left and right antenna at the *t*-th search, and *d^t^* is the sensing length of the antenna: the larger the sensing length, the stronger the searching ability of the beetle. Initially, the sensing length of the antennae is long enough to cover a suitable search area to jump out of the local minimum; then, it gradually decays over time. The information intensities detected by the left and right antenna are judged according to the fitness function, and the position of the beetle is updated:

(32) $$x^{t+1}=x^{t}+\lambda^{t}\cdot \vec{S}\cdot sign[f(x_{rt})-f(x_{lt})]$$

where *x^t+^*^1^ is the space position of the beetle at *t*+1-th search, *λ^t^* is the step size of the beetle’s movement during the *t*-th search, *sign*(.) is a symbolic function, and *f*(.) is the fitness function. In addition, the updating rules of *d^t^* and *λ^t^* can be referred to as follows [31]:

(33) $$\begin{array}{l} d^{t+1}=0.95d^{t}+0.01\\ \lambda^{t+1}=0.95\lambda^{t} \end{array}$$

#### 3.4.3. Elman Neural Network (Elman NN)

**Figure 6:**
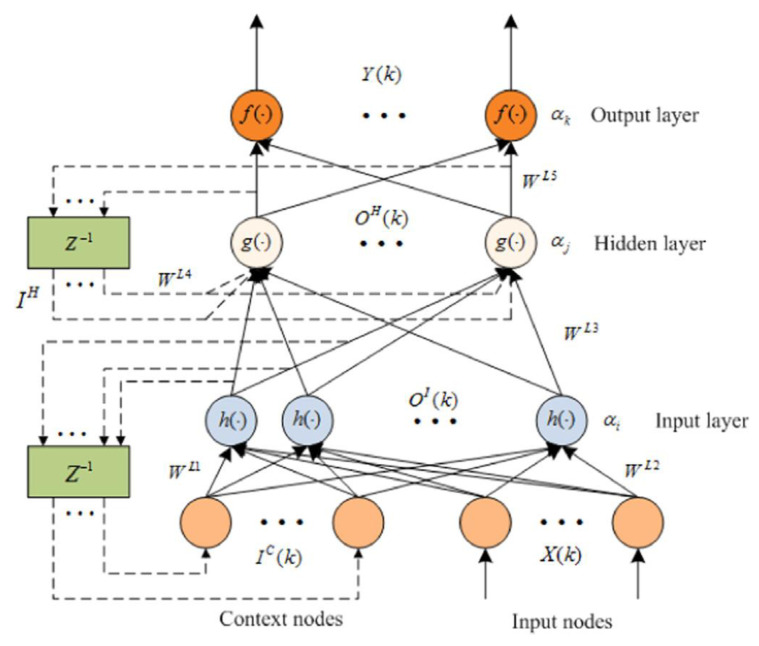
The structure of the Elman neural network.

The Elman NN is a typical dynamic recursive network proposed by Elman; compared with the three-layer structure of the BP neural network, the input layer of the Elman neural network contains context nodes, whose function is to remember the previous activation of hidden layer nodes, which makes the network have the adaptability of time-varying characteristics and thus increases the global stability of the network. The structure of the Elman NN is given in Figure 6 [22].

Referring to the network structure of the Elman NN in Figure 6, the relationship between input and output is given [22]:

(34) $$\{\begin{array}{c} O^{I}(k)=h(W^{L1}I^{C}(k)+W^{L2}X(k)-a_{i})\\ \begin{array}{l} O^{H}(k)=g(W^{L3}O^{I}(k)+W^{L4}I^{H}(k)-a_{j})\\ Y(k)=f(W^{L5}O^{H}(k)-a_{k})\\ I^{C}(k)=O^{I}(k-1),I^{H}(k)=O^{H}(k-1) \end{array} \end{array}$$

where *X*(*k*) and *Y*(*k*) are the input layer and output layer vectors, respectively, *W^L^*^1^, *…*, *W^L^*^5^ are the connection weights, *a_i_*, *a_j_*, and *a_k_* are the thresholds of each layer, *h*(·), *g*(·), and *f*(·) are the activation functions, and the activation functions *h*(*x*) and *g*(*x*) adopt a sigmoid function:

(35) $$f(x)=g(x)=\frac{1}{1+e^{-x}}$$

#### 3.4.4. Elman Neural Network Based on Beetle Antennae Search Algorithm

Similar to the BP neural network, the initial weights and thresholds of the Elman NN are generated randomly, which may lead to problems such as local optimum and slow speed in the search process. This paper adopts the BAS algorithm to optimize the weights and thresholds of the Elman NN, and the steps of the BAS–Elman NN algorithm are as follows:

1. According to the structure of the Elman NN, the dimension of the BAS search space is determined.

2. Create the vector of the beetle antennae’s orientation and the position of the center of mass.

3. Determine the fitness function for evaluation, and take the mean square error (MSE) of the expected and predicted output of training data as the fitness function:

(36) $$fitness=\frac{1}{N}\sum_{i=1}^{N}(y_{p,i}-y_{e,i}{)}^{2}$$

where *N* is the number of samples used for training, and *y_p,i_* and *y_e,i_* are the predicted value and expected value of the *i*th sample, respectively. When the iteration of the BAS algorithm terminates, the solution space vector with the smallest fitness value is the optimal solution.

4. The spatial positions of the left and right antennae are calculated, and the fitness function values of the left and right antennae are compared to update the position of the beetle.

5. Determine whether the stop condition is met. If not, return to Equation (4) and continue iterating.

### 3.5. Parallel Processing Model Based on MOVMD–TFPF and BAS–Elman NN

**Figure 7:**
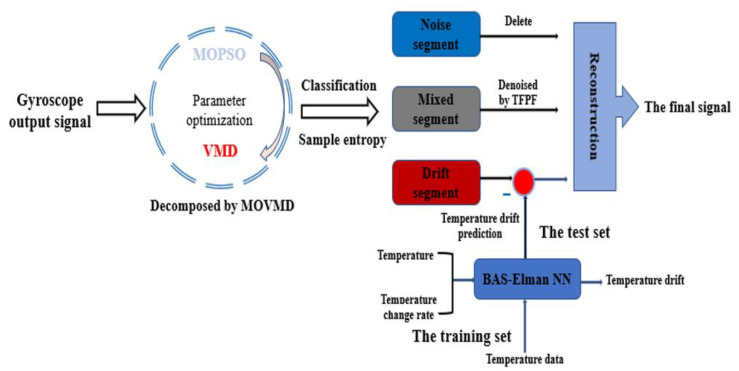
Parallel processing model based on MOVMD–TFPF and BAS–Elman NN.

After introducing the above algorithms, the parallel processing model for eliminating noise and temperature drift based on MOVMD–TFPF and BAS–Elman NN is put forward. Figure 7 shows the processing process of the parallel processing model, and the main steps are as follows.

Step 1. The temperature change and the corresponding gyroscope output are obtained after the temperature experiment. Firstly, multi-objective particle swarm optimization is adopted to determine the optimal decomposition parameters [*k*_best_,*a*_best_] of the VMD, and the multi-objective optimized VMD (MOVMD) is obtained. Then, the output signal is decomposed by MOVMD, and some intrinsic mode functions (IMFs) can be obtained.

Step 2. Secondly, the sample entropy is adopted to classify the obtained IMFs into a noise segment, mixed segment, and drift segment.

Step 3. For the noise segment, it belongs to high-frequency noise and does not contain temperature trend and useful signals, so it can be directly discarded. For the mixed segment, it contains noise and useful components, so TFPF is used for denoising to obtain useful components. Then, BAS-Elman NN is trained by using the temperature and temperature change rate of the training set as the input and temperature drift as the output, and it is applied to predict the drift component in the drift segment to achieve the purpose of compensation.

Step 4. Finally, the denoised mixed segment and compensated drift segment are reconstructed to form the final gyroscope output.

## 4. Experiment and Analysis

### 4.1. The Experimental Process

**Figure 8:**
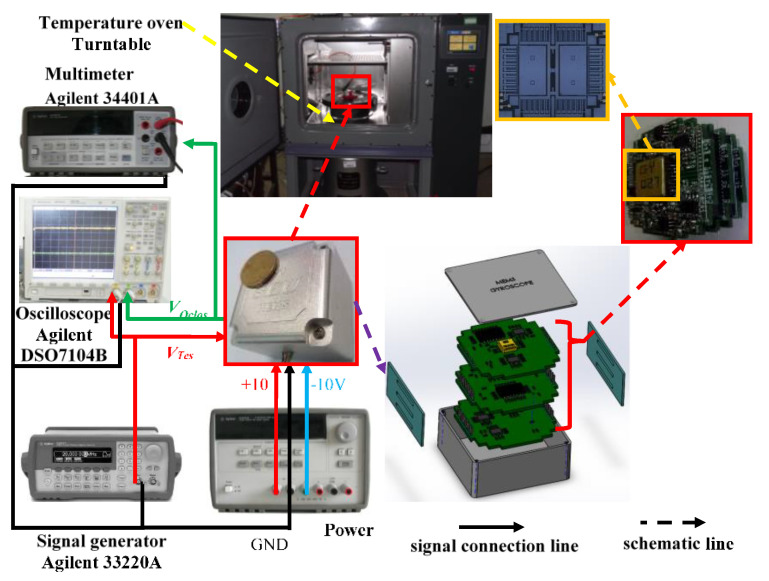
Gyroscope temperature test equipment.

**Figure 9:**
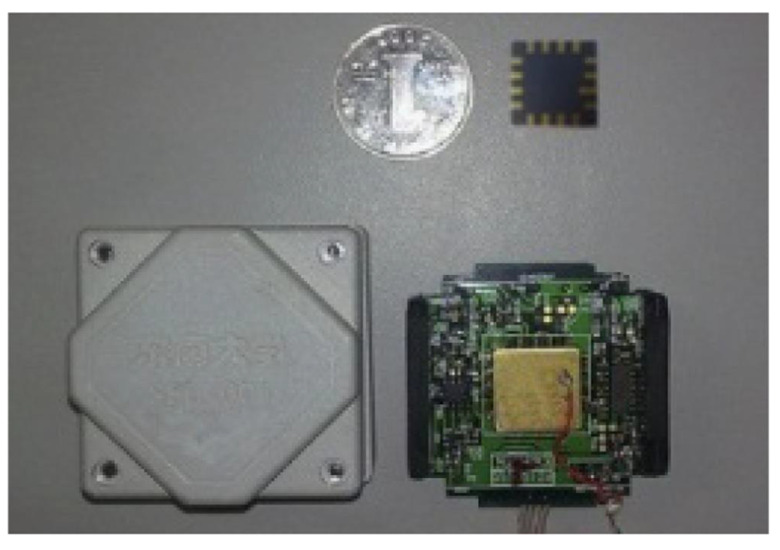
The PCB and gyroscope overall photos.

To certify the superiority of the parallel processing algorithm proposed in this paper, a gyroscope temperature output experiment is carried out. Figure 8 shows the experimental equipment, and Figure 9 shows the structure package and circuit of the gyroscope. A detection circuit is designed in three PCB boards, respectively; the metal pins not only connect the electronic signals of the detection circuit but also can be used to connect three PCBs, which are wrapped with a rubber pad. In addition, after the PCB is wrapped with a rubber pad, it is placed in the metal shell; such doing can reasonably and effectively guard the structure of the chip, and it also can avoid severe impact on PCB. To avoid the interference of the electromagnetic field, the ground signal and the metal shell are connected. In addition, one of the above three PCBs is connected to the structural chip as a weak signal interface, and the other two PCB circuits play the role of induction circuit and drive circuit, respectively.

The experimental equipment is fitted with a temperature-controlled oven (Agilent 34401 A, Agilent, Santa Clara, CA, USA), a multimeter, a signal generator (Agilent 33220 A), and a DC power supply of ± 10 V (Agilent E3631 A).

The experimental procedure is as follows. First, we turned on the gyroscope and left it at room temperature for one hour. Later, we quickly heated the temperature-controlled oven to 60 °C to achieve the purpose of making the temperature in the gyroscope housing consistent with the controlled temperature (60 °C), and we maintained it in this state for one hour. When the temperature control oven dropped 10 °C, the gyroscope remained working in this state for one hour, and when the temperature decreased to –40 °C, the gyroscope continued working for the last hour, and then the experiment ended. It should be noted that two groups of experimental data were collected in this paper to train and test the model, and the subsequent algorithm demonstration and comparative analysis are based on the test set. The temperature controlling system precision of the oven is 0.1 °C, and this work employed a temperature sensor to detect the temperature information inside the gyroscope and make sure the gyroscope output signal and temperature value are collected synchronously.

### 4.2. The Experimental Results

**Figure 10:**
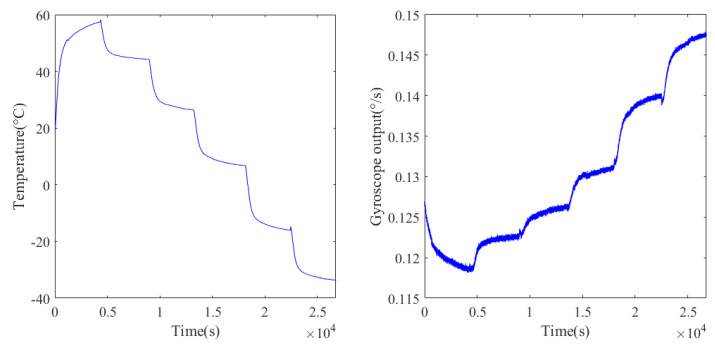
Experimental temperature change and the corresponding gyroscope output.

The experiment results are given in Figure 10; in the experiment, the temperature varies from 60 to –40 °C with time, and the gyroscope output contains rich noise; meanwhile, the output drifts with the temperature, and the influence of drift makes the output of the gyroscope change from 0.115° to 0.15°. Since the output of the gyroscope is accompanied by drift and noise, a parallel processing model is needed to eliminate these influences.

**Figure 11:**
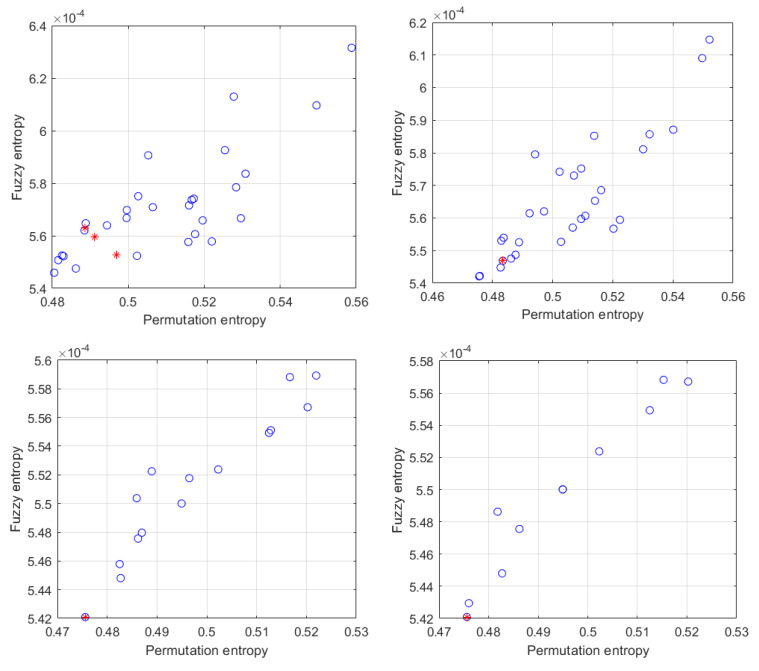
The result of particle convergence during the iterative process.

According to the algorithm steps, the experimental signals need to be decomposed by VMD firstly, and the optimal parameters [*k*_best_,*a*_best_] need to be determined by MOPSO before decomposition. The parameters for MOPSO are set as follows: *Np* and *N_R_* are set at 40, the maximum iteration number *M* is set at 10, the inertia weight *W* is set at 0.4, while the learning factors *c*_1_, *c*_2_ are both set at 1.5. The optimization range of parameter *k* and *a* are set as [4, 12] and [1000, 10000], respectively. The convergence evolution of particles is shown in Figure 11, where the Pareto front optimal solution set is marked in red, and the optimal decomposition parameter [*k*_best_, *a*_best_] = [8, 9755].

**Figure 12:**
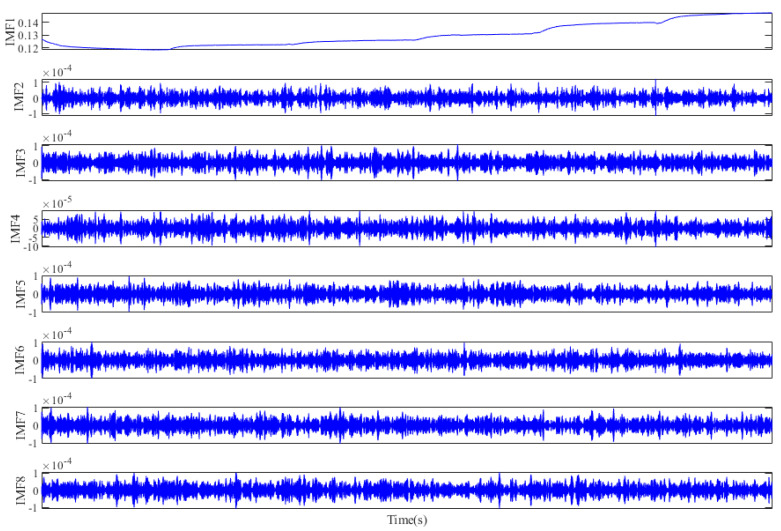
The decomposition of gyroscope output by MOVMD.

**Figure 13:**
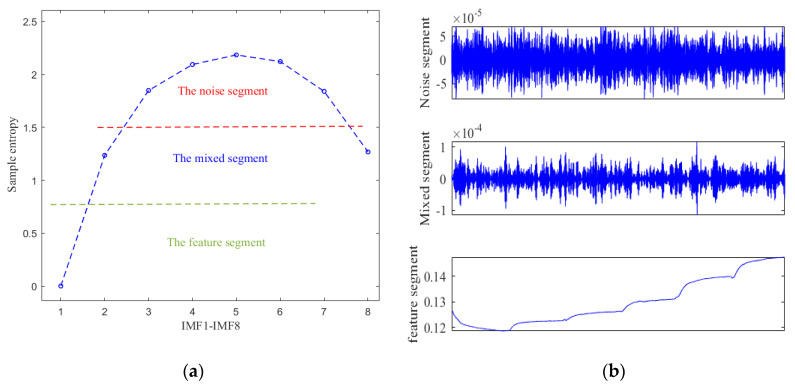
(**a**) Sample entropy of each IMF; (**b**) Classification results of IMFs.

After decomposition, the eight-layer IMFs are obtained, and it is shown in Figure 12 that there is an obvious trend of temperature drift in IMF1, and it is difficult to distinguish whether IMF2–IMF8 belong to noise or the mixture of drift and noise. So, the sample entropy [41] is adopted to distinguish these IMFs. Figure 13a,b respectively show the sample entropy of each IMF and the classification results, and the IMFs are divided into a drift segment, mixed segment, and noise segment.

Subsequently, the parallel processing model is implemented. For the noisy segment, it is considered as a useless signal and can be discarded directly. For the mixed segment, it is a mixture of noise and drift caused by temperature changes. Therefore, TFPF is adopted here to denoise the mixed segment to preserve useful components. For the drift segment, it is mainly the drift caused by the temperature change, showing a nonlinear change trend. In this paper, the temperature compensation model based on the BAS-Elman NN is used to deal with it.

**Figure 14:**
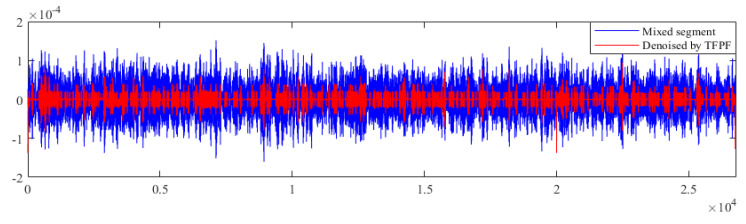
The comparison of mixed segments before and after denoising by TFPF.

**Figure 15:**
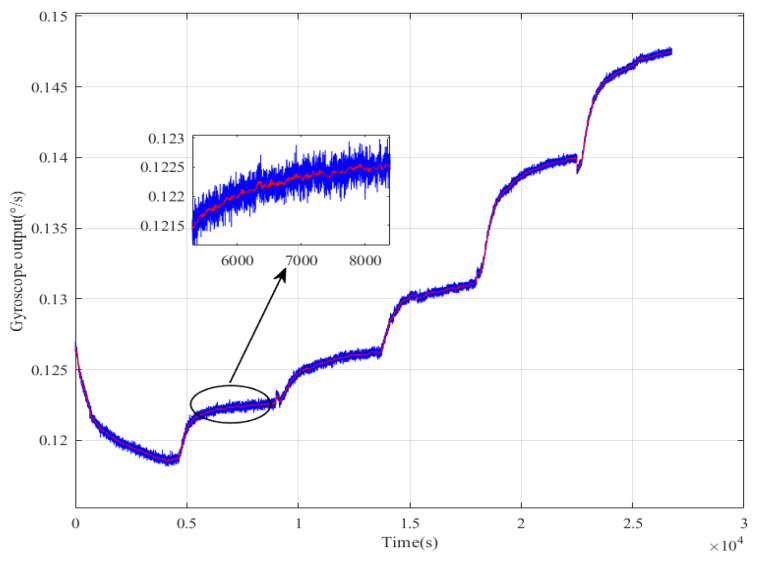
The comparison of gyroscope output signals before and after denoising.

Figure 14 shows the comparison of mixed segments before and after denoising by TFPF; then, the denoised gyroscope output signal can be obtained by reconstructing the denoised mixed segment and the uncompensated drift segment. In Figure 15, the noise in the gyroscope output signal is basically eliminated.

**Figure 16:**
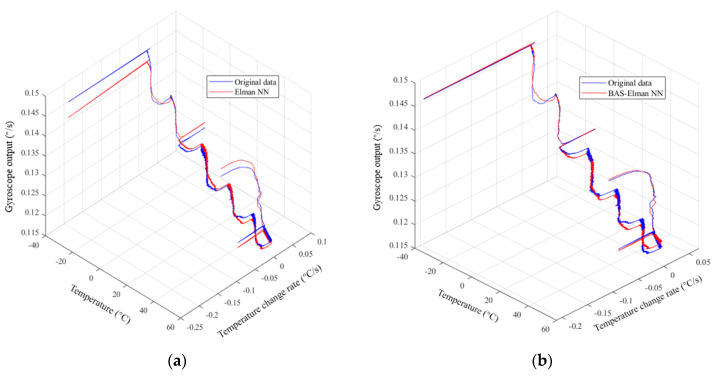
(**a**) Comparison of predicted output and actual output of Elman NN before optimization; (**b**) Comparison of the optimized predicted output and actual output of Elman NN.

**Figure 17:**
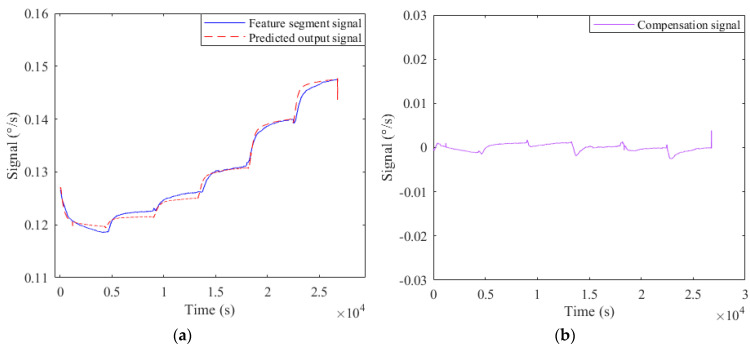
(**a**) Predicted output of drift segment; (**b**) Compensation result of drift segment.

Before using the Elman NN for temperature compensation, the beetle antenna search algorithm is adopted to optimize the network weights of Elman NN to improve its learning accuracy. Figure 16a,b are the comparison of predicted output of Elman NN before and after optimization and real output respectively; it can be clearly seen that the prediction accuracy of the optimized Elman NN has been significantly improved. Figure 17a is the real output of the drift segment and the predicted output based on temperature and temperature change rate; Figure 17b is the compensation result of the drift segment.

**Figure 18:**
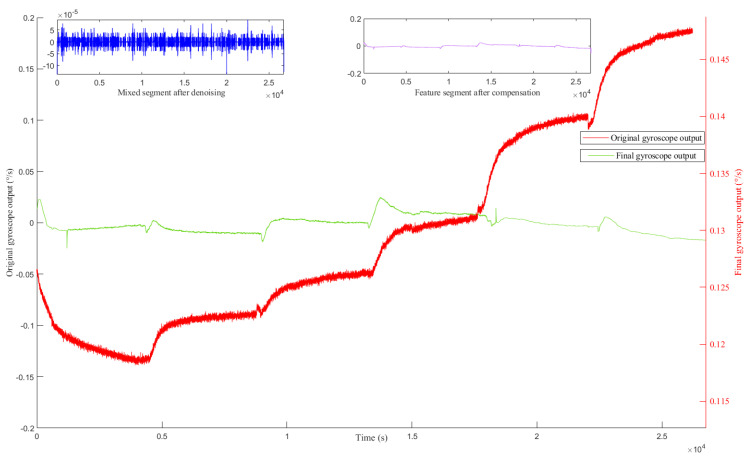
The final gyroscope output signal after denoising and compensation.

Then, the denoised mixed segment and the compensated drift segment are reconstructed to form the final gyroscope output; the noise and temperature drift in the gyroscope output signal have been well eliminated in Figure 18.

**Figure 19:**
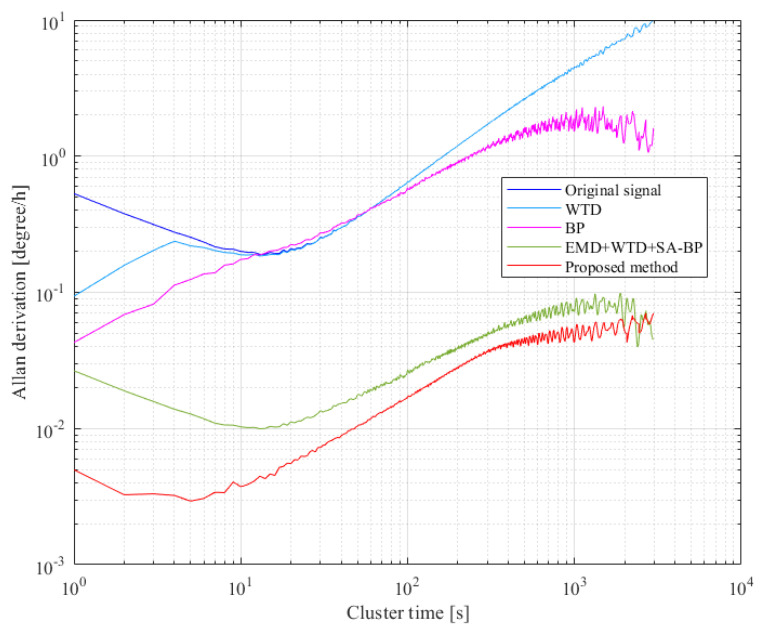
Allan variance analysis of the output signals before and after processing.

In order to further highlight the superiority of the proposed compensation scheme, we compare it with the wavelet threshold denoising (WTD), the BP neural network, and the advanced temperature compensation algorithm that combines the empirical mode decomposition (EMD), wavelet threshold denoising (WTD), and simulated annealing optimized BP neural network. As an error analysis method in IEEE standard [42], Allan variance analysis is widely used in the stochastic error modeling of inertial devices. In this paper, the angle random walk and bias stability of the original gyroscope output and the compensated gyroscope output are quantitatively analyzed by Allan variance analysis. Comparison results are given in Figure 19; the accuracy of the final signal obtained by direct denoising or compensating the gyroscope output signal is very low. Even compared with the advanced EMD + WTD + SA-BP algorithm, the proposed scheme is still superior. After denoising and compensation of the proposed scheme, the angle random walk of the original gyroscope output decreases from 0.531076 to 5.22502 × 10^−3^°/h/√Hz, and the bias stability is reduced from 32.7364°/h to 0.140403°/h; this proves the superior performance of the proposed parallel processing model.

## 5. Conclusions

In this paper, a parallel processing model based on MOVMD–TFFPF and BAS–Elman NN is established to eliminate the noise and temperature drift in the MEMS gyroscope’s output signal. Firstly, the multi-objective particle swarm optimization is adopted to determine the optimal decomposition parameters of the VMD, and the multi-objective optimized VMD (MOVMD) is obtained. After MOVMD decomposition and SE classification, the gyroscope output signal is divided into a noise segment, mixed segment, and drift segment; then, the noise segment is discarded directly, and the mixed segment is denoised by TFPF. Subsequently, the double-input/single-output temperature compensation model based on the BAS-Elman NN is established to compensate the drift segment. Finally, the denoised mixed segment and compensated drift segment are reconstructed to form the final gyroscope output, and the experimental results and comparative analysis verify the validity of the parallel processing model.
